# Significant Increase in C-Reactive Protein and Serum Amyloid A in the Early Hours of Paroxysmal Atrial Fibrillation

**DOI:** 10.14740/cr455w

**Published:** 2016-02-20

**Authors:** Mariya Negreva, Svetoslav Georgiev, Krasimira Prodanova

**Affiliations:** a First Clinic of Cardiology, Varna University Hospital “St. Marina”, Bulgaria; b Second Clinic of Cardiology, Varna University Hospital “St. Marina”, Varna, Bulgaria; c Faculty of Applied Mathematics and Informatics, Technical University of Sofia, bul. Kl. Ohridski 8, Sofia, Bulgaria

**Keywords:** Inflammation, Paroxysmal atrial fibrillation, Sinus rhythm

## Abstract

**Background:**

A number of data have been accumulated on inflammation in persistent and permanent atrial fibrillation (AF). Our aim was to study the process in paroxysmal AF (PAF) by measuring plasma concentrations of high-sensitivity C-reactive protein (hs-CRP), serum amyloid A (SAA) and fibrinogen in dynamics.

**Methods:**

The markers were investigated in 51 patients (26 males and 25 females; 59.84 ± 1.60 years) at hospital admission (baseline), 24 hours and 28 days after sinus rhythm restoration. Fifty-two controls (26 males and 26 females; 59.50 ± 1.46 years) were selected.

**Results:**

At baseline, hs-CRP and SAA concentrations were higher in patients (8.12 ± 0.82 vs. 5.57 ± 0.21 mg/L, P = 0.003; 16.04 ± 0.93 vs. 5.12 ± 0.23 ng/mL, P < 0.001, respectively) and these changes persisted 24 hours after sinus rhythm restoration (8.16 ± 0.71 vs. 5.57 ± 0.21 mg/L, P < 0.001; 12.99 ± 0.75 vs. 5.12 ± 0.23 ng/mL, P < 0.001, respectively). On the 28th day, no significant difference was measured (5.42 ± 0.29 vs. 5.57 ± 0.21 mg/L, P = 0.68; 5.89 ± 0.38 vs. 5.12 ± 0.23 ng/mL, P = 0.08, respectively). At any measurement, fibrinogen levels did not differ between patients and controls (3.30 ± 0.17 vs. 3.22 ± 0.11 g/L, P = 0.70; 3.32 ± 0.11 vs. 3.22 ± 0.11 g/L, P = 0.52; 3.24 ± 0.13 vs. 3.22 ± 0.11 g/L, P = 0.90, respectively).

**Conclusion:**

PAF is associated with dynamics in hs-CRP and SAA plasma levels. The results suggest that inflammation is closely related to the arrhythmia initiation.

## Introduction

In recent years, a number of data have been accumulated on inflammatory process in atrial fibrillation (AF). Histological studies revealed inflammatory changes in the atrial myocardium [1, 2]. Elevated values of chemokines, interleukins, acute phase proteins and others were measured in patients with AF [3, 4]. Scientific interest has mostly focused on persistent and permanent AF. Data on inflammation in paroxysmal atrial fibrillation (PAF) are scarce.

The systemic response to inflammation, which occurred in human organism, is in its essence a combination of pathophysiological and biochemical changes aiming to limit the harmful effects (inflammatory stimuli) and to quickly recover homeostasis [5]. They follow both acute and chronic inflammation [6].

One of the most important features of the inflammatory response is the changes in plasma concentrations of a number of proteins known as acute phase proteins (APPs). They are those whose blood levels change by at least 25% in the course of an inflammatory disorder [7]. The degree of change is proportional to the amount of inflammation and thus they can provide a good opportunity for monitoring the inflammatory process [8].

At present, the group of APPs includes about 40 proteins that are characterized by an extreme structural and functional diversity, e.g. components of the complement system, coagulation and fibrinolytic system, antiproteases, transport proteins and the like. This naturally and logically predetermines the significant differences in the response of each protein, namely the onset and intensity of synthesis under the influence of inflammatory stimulus.

C-reactive protein (CRP) is one of the major APPs. It is extremely sensitive and at the same time a non-specific systemic marker of inflammation and tissue damage [9]. CRP has a relatively short half-life of about 19 h and the plasma concentrations primarily depend on the intensity of hepatic synthesis. They rise around the 10th hour after the inflammatory stimulus and reach a peak during the second day. The levels of acute phase proteins in the blood are characterized by rapid dynamics, reflecting the changes in the strength of the inflammatory response [10]. Recently, a preferred marker of inflammation has been high-sensitivity CRP (hs-CRP) because hs-CRP assay is designed to measure very low levels of CRP [11].

Another important APP for inflammation is serum amyloid A (SAA) that is synthesized in large quantities in the liver [12]. SAA has a high sensitivity to inflammatory events and its levels can increase up to 1,000-fold [13, 14]. Like CRP, changes in plasma concentrations occur early after the onset of inflammation and then rapid decline is observed in the attenuation of the process [7]. Hs-CRP and SAA give immediate information about the changes in the inflammatory activity. The two proteins are preferred markers in the assessment of inflammation [15].

Fibrinogen, like CRP and SAA, is an APP. In contrast, it has a long plasma half-life (about 100 h) and its levels remain high a couple of days after the application of the inflammatory stimulus. Therefore, the study of this indicator can give us information about previous inflammatory activity.

On the other hand, in the course of the inflammatory process, its plasma levels increase later and slower compared to hs-CRP and SAA, and can be measured as normal in case of a recently applied inflammatory stimulus.

The characteristics of the changes in the plasma levels of hs-CRP, SAA and fibrinogen enable these indicators to complement each other in the study of inflammatory response.

The purpose of the study was to study the inflammatory response in PAF by measuring plasma concentrations of the APPs hs-CRP, SAA and fibrinogen in dynamics.

## Materials and Methods

### Study design

Patients with an occurrence of PAF episodes < 48 h prior to the time of hospitalization were screened for the study. A criterion for determining the time span of rhythm disorder was the patients’ detailed case history, in which they clearly determined the beginning of the episode of AF as a subjective sensation of “palpitation”, persisting until hospitalization. The diagnosis “AF” was accepted after its objectification by electrocardiographic examination, performed immediately after admission to the ward. In the absence of contraindications to the use of propafenone, described elsewhere [16, 17], the drug was used for sinus rhythm restoration. The patients who recovered sinus rhythm were observed until the 28th day after the farmacoversion that was the end of the study.

Only patients with no AF recurrence until the end of the study after the initial restoration of sinus rhythm were selected. During the study period, patients were screened for AF by ECG monitoring. They were constantly monitored by ECG during their hospital stay, i.e. 24 h after restoration of sinus rhythm. Subsequently, two control check-ups were performed, on the seventh and 28th day after the farmacoversion, during which 24-h Holter monitoring was made and a detailed history of recurrence of “palpitation” or similar sensation was taken. The asymptomatic forms of AF were no subject of our study.

A control group was formed which included participants without a history or electrocardiographic evidence of AF. The controls were selected and screened from healthy volunteers, who visited their doctor for prophylactic annual examinations.

The health status of all participants in the study was determined based on history, medical records, physical examination, laboratory tests and repeatedly performed electrocardiograms and transthoracic echocardiography.

The plasma concentrations of hs-CRP, SAA and fibrinogen were examined in all study participants.

In the patient group, blood samples were taken three times: immediately after hospitalization (baseline values), 24 h and 28 days after the restoration of sinus rhythm. The control subjects were tested once.

The study was conducted in the ICU of the First Cardiology Clinic at the University Hospital “St. Marina”, Varna for the period from October 2010 to May 2012 after the approval by the Ethics Committee of Scientific Research (no. 35/29.10.2010) at the same hospital and in accordance with the Declaration of Helsinki [18]. The participants in the study were included after previously signing the informed consent requirements.

### Study population and patient selection

From a total of 338 screened patients with PAF, only 56 participants were selected (31 men and 25 women) with restored and permanently retained sinus rhythm until the end of the study without the presence of recurrences. Two hundred eighty-two patients with PAF were dropped out of the study due to exclusion criteria (see exclusion criteria).

To balance the gender structure of the patient group, 51 patients were consecutively selected (26 men and 25 women) with a mean age of 59.84 ± 1.60 years.

Exclusion criteria included the following: 1) cardiovascular diseases including ischemic heart disease, heart failure, congenital or inflammatory heart diseases, moderate or severe acquired valvular defects, and cardiomyopathies; 2) other diseases including renal, hepatic or pulmonary failure, central nervous system diseases, inflammatory and/or infectious diseases during the past 3 months, neoplastic or autoimmune diseases, and diseases of the endocrine system (excluding diabetes mellitus type 2, good control); 3) hormone replacement therapy, pregnancy, systemic administration of analgesics including NSAIDs, and obesity with body mass index (BMI) > 35; 4) persisting of the rhythm disorder after propafenone application, restoration of sinus rhythm by electrical cardioversion, and recurrence of AF by the end of the study (exclusion criteria for patients); 5) contraindications to treatment with propafenone (exclusion criteria for patients).

The same exclusion criteria were applied (see above) for the formation of the control group. Thus, the selection of participants (patients and controls) was aimed to a maximum degree to eliminate or equalize the factors that influence inflammation in both groups. From a total of 169 screened, 52 were selected as controls for the study. Their average age was 59.50 ± 0.46 years, and the men and women have an equal number of 26 (50%).

### Blood samples and laboratory tests

APPs were investigated in venous blood obtained after puncture of a peripheral vein. The blood necessary for measuring the plasma concentrations of hs-CRP and SAA was collected in a heparin tube (VACUETTE/4.0 mL/Li Hep). A coagulation tube with a buffered sodium citrate solution (VACUETTE/2.0 mL/sodium citrate 3.2%) was used to study the levels of fibrinogen. Centrifuging of the samples and storage of resulting plasma were carried out in full accordance with the methodology used.

Plasma levels of hs-CRP were determined by immunoturbidimetric methodology, using a test for sensitive determination of CRP (HUMAN Gesellschaft fur Biochemica und Diagnostica mbH, Wiesbaden, Germany). Plasma concentrations of SAA were measured by ELISA kit (Elabscience Biotechnology Co., Ltd, China). The levels of fibrinogen were investigated with a test by Diagnostica Stago Inc., Mannheim. The lower limits of detection were 0.1 mg/L for hs-CRP, 1.25 ng/mL for SAA and 1.5 g/L for fibrinogen. The intra-assay and inter-assay coefficients for the markers were < 5%. Laboratory procedures were completely done in accordance with the manufacturers’ protocols.

All samples were tested twice, taking the average value of the measurements into account when calculating the results.

Re-freezing of the samples was not allowed during the study.

### Propafenone regimen

Propafenone was administered according to the prescribed scheme: IV 2 mg/kg bolus followed by infusion at a dose of 0.0078 mg/kg/min for 120 min and p.o. administration at a dose of 300 mg three times at intervals of 8 h. In case of restoration of sinus rhythm, the scheme was discontinued, and until the end of the study, all patients received a maintenance dose of propafenone - p.o. 150 mg three times daily. All patients were under constant monitoring until hospital discharge, i.e. 24 h after restoration of sinus rhythm.

### Statistical analysis

The means, relative shares and central tendency (Mo = mode) were calculated using descriptive statistics. The testing of the equality hypothesis was done using Student’s *t*-test. Data analysis was performed with a specialized statistical package GraphPad PRISM, Version 5.00. The results were presented as mean ± standard error of the mean (SEM) or n (%). Values of P < 0.05 were considered statistically significant.

The predictive value of hs-CRP, SAA and fibrinogen for PAF manifestation was determined by logistic regression analysis.

## Results

### Baseline patient characteristics

Demographics, clinical characteristics and medical treatment of patients’ and control group are presented in Tables 1, 2 and 3. There were no significant differences between the two groups in any of the following variables (P > 0.05): number of participants, mean age and gender structure (Table 1), as well as accompanying diseases, dyslipidemia and ongoing treatment (until the time of hospitalization) (Table 2).

**Table 1:** Demographic Characteristics of Participants

	Patients with PAF	Control group	P values
Number of participants in the group	51	52	0.89
Mean age (years)	59.84 ± 1.60	59.50 ± 1.46	0.87
Men/women	26/25	26/26	1/0.93

**Table 2:** Clinical Characteristics of Patients and Control Subjects

	Patients with PAF	Control group	P values
Accompanying diseases			
Hypertension	37 (72.54%)	34 (65.38%)	0.44
Diabetes mellitus type 2	3 (5.88%)	2 (3.84%)	0.62
Chronic ulcer disease	2 (3.92%)	0	0.15
Status after hysterectomy	2 (3.92%)	1 (1.92%)	0.54
Benign prostatic hypertrophy	1 (1.96%)	0	0.32
Dyslipidemia	4 (7.84%)	3 (5.77%)	0.69
Medicaments for hypertension and dyslipidemia			
Beta blockers	19 (37.25%)	17 (32.69%)	0.62
ACE inhibitors	15 (29.41%)	14 (26.92%)	0.78
Sartans	11 (21.57%)	9 (17.31%)	0.58
Statins	4 (7.84%)	3 (5.77%)	0.69

**Table 3:** Deleterious Habits and BMI in Patients and Controls

	Patients with PAF	Control group	P values
Deleterious habits			
Smoking	8 (15.69%)	7 (13.46%)	0.75
Alcohol intake	7 (13.72%)	6 (11.53%)	0.74
BMI (kg/m^2^)	23.85 ± 0.46	24.95 ± 0.45	0.09

The groups also had no statistically significant difference in frequency of bad habits and BMI (P > 0.05) (Table 3).

The performed statistical analysis of the time of the occurrence of AF until hospitalization showed that all 51 patients were hospitalized between the second and the 24th hour after the onset of the arrhythmia, and most frequently during the fifth hour (Mo = 5, for 10 of all 51 patients). The mean duration of the AF episodes until hospitalization was 8.14 ± 0.76 h.

### Plasma concentrations of APPs

**Figure 1:**
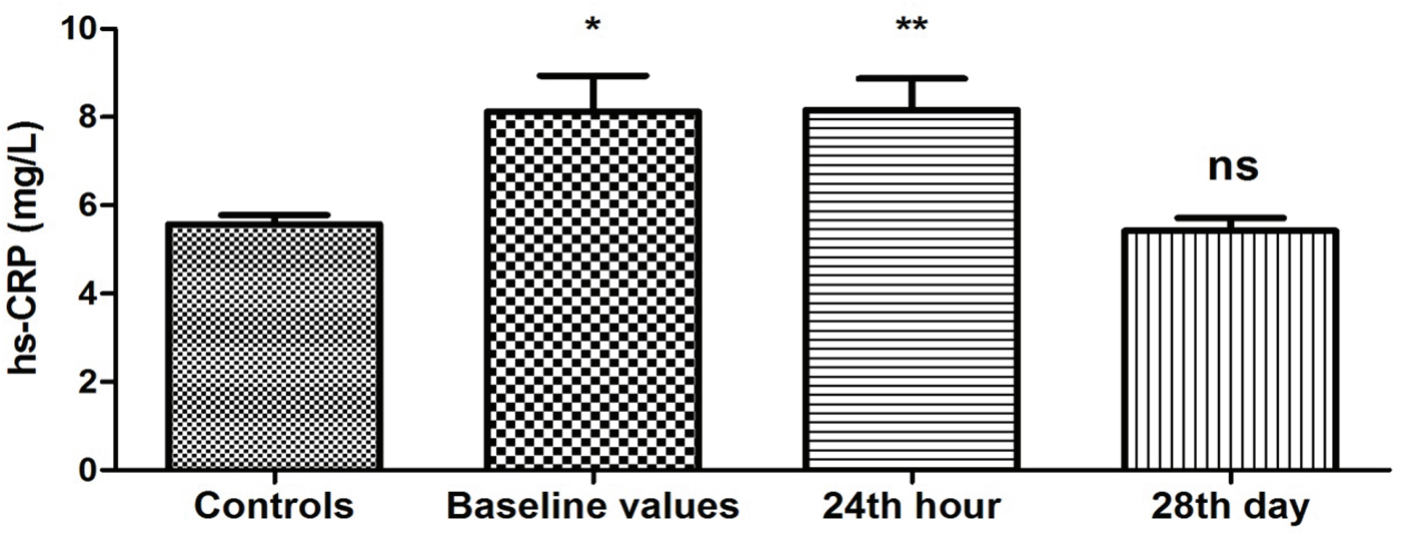
Changes in plasma concentration of hs-CRP (mg/L) in patients with PAF. Baseline values: hs-CRP values upon patients’ hospitalization; 24th hour: values 24 h after sinus rhythm restoration; 28th day: values 28 days after sinus rhythm restoration; *P < 0.05; **P < 0.001; ns: statistically insignificant difference.

The results obtained from the study of plasma levels of hs-CRP are presented in Figure 1. It shows that hs-CRP concentrations were significantly higher at baseline in PAF group compared to the controls (8.12 ± 0.82 vs. 5.57 ± 0.21 mg/L, P = 0.003). Twenty-four hours after restoration of sinus rhythm, the changes persisted (8.16 ± 0.71 vs. 5.57 ± 0.21 mg/L, P < 0.001). On the 28th day, hs-CRP levels in PAF group were not statistically different compared to the control group (5.42 ± 0.29 vs. 5.57 ± 0.21 mg/L, P = 0.68).

**Figure 2:**
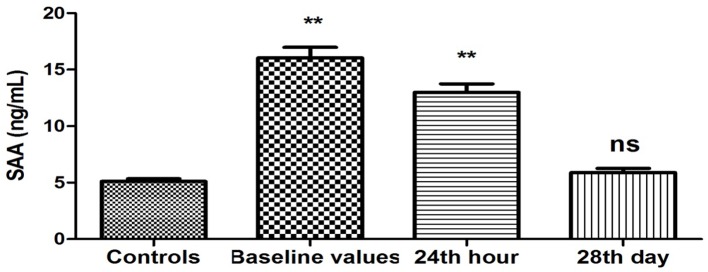
Changes in plasma concentration of SAA (ng/mL) in patients with PAF. Baseline values: hs-CRP values upon patients’ hospitalization; 24th hour: values 24 h after sinus rhythm restoration; 28th day: values 28 days after sinus rhythm restoration; **P < 0.001; ns: statistically insignificant difference.

Plasma levels of SAA were elevated (16.04 ± 0.93 vs. 5.12 ± 0.23 ng/mL, P < 0.001) upon patient’s admission to the ward compared to the controls (Fig. 2). This difference was maintained on the 24th hour after restoration of sinus rhythm (12.99 ± 0.75 vs. 5.12 ± 0.23 ng/mL, P < 0.001). Twenty-eight days after the farmacoversion, there was no statistically significant difference compared to controls (5.89 ± 0.38 vs. 5.12 ± 0.23 ng/mL, P = 0.08).

**Figure 3:**
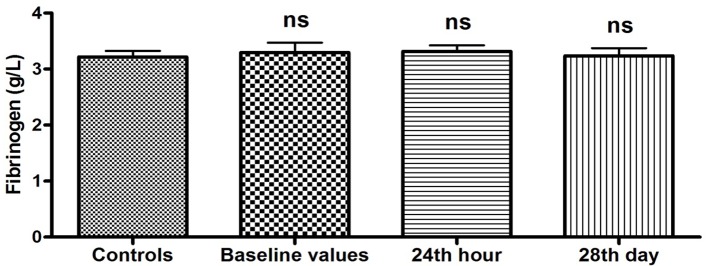
Plasma levels of fibrinogen (g/L) in patients with PAF. Baseline values: hs-CRP values upon patients’ hospitalization; 24th hour: values 24 h after sinus rhythm restoration; 28th day: values 28 days after sinus rhythm restoration; ns: statistically insignificant difference.

There was no statistically significant difference in the plasma levels of fibrinogen between controls and patients at hospitalization (baseline values), and 24 h and 28 days after the restoration of sinus rhythm (3.30 ± 0.17 vs. 3.22 ± 0.11 g/L, P = 0.70; 3.32 ± 0.11 vs. 3.22 ± 0.11 g/L, P = 0.52; 3.24 ± 0.13 vs. 3.22 ± 0.11 g/L, P = 0.90, respectively) (Fig. 3).

**Figure 4:**
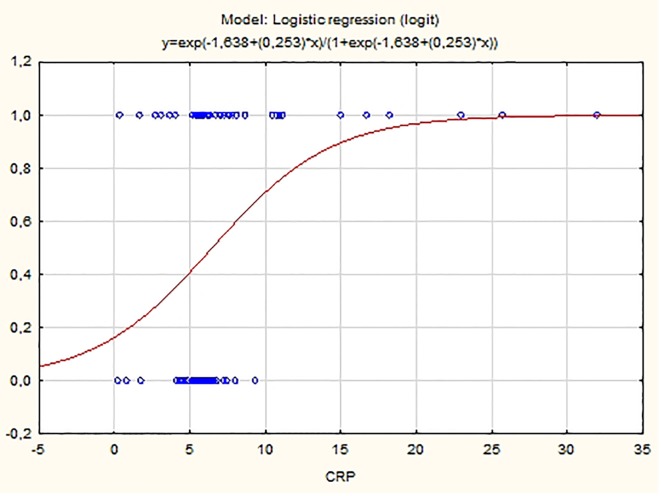
Probability distribution of hs-CRP estimated using logistic model.

Logistic regression model with single explanatory variable shows that plasma concentrations of hs-CRP are a statistically significant predictor for PAF manifestation at the level of significance 0.05 (P = 0.01). The fitted logistic model has the equation:

ln(P/(1 - P)) = -1.638 + 0.253 hs-CRP

where P is the probability for the occurrence of PAF. As the APP value increases, so does the probability P for the presence of PAF (β_1_ = 0.253 > 0) (Fig. 4). The logistic model correctly classifies 62.14% of the observed cases in our sample (OR: 3.34, 95% CI: 1.05 - 5.15).

Plasma concentrations of SAA are also a statistically significant predictor for the arrhythmia appearance (P < 0.001). The fitted logistic model has the equation:

ln(P/(1 - P)) = -6.675 + 0.812 SAA

where P is the probability for the occurrence of the PAF. As SAA value increases, the probability for PAF manifestation also increases (β_1_ = 0.812 > 0).

Plasma levels of fibrinogen have no predictive value for PAF (P > 0.05).

## Discussion

The results of our study show a unidirectional and significant change in the baseline values of two of the studied APPs, namely hs-CRP and SAA (Figs. 1, 2). The plasma concentrations of both indicators were significantly elevated compared to those of the controls (P < 0.05). It is known that hs-CRP and SAA are reliable inflammatory biomarkers [10, 15]. Their high values are indicative of an enhanced inflammatory response due to increased inflammatory activity in the human organism. In this sense, our results give us reason to assume that there is a significant increase in the APPs hs-CRP and SAA as a reaction to the presence of inflammation in the clinical manifestation of PAF.

It is appropriate to note that the APPs have been studied between the second and 24th hour (most often in the fifth hour) after the manifestation of AF. It is for the first time that elevated levels of hs-CRP and SAA are measured in these early hours of the rhythm disorder (on the average in the eighth hour). This fact could be a logical explanation for the lack of change in the values of fibrinogen, given its slow dynamics in plasma levels. At the same time, the early changes in the concentrations of hs-CRP and SAA give serious grounds to suppose that they are closely related to the initiating mechanisms of the disease. The idea of the involvement of the inflammatory process in the pathogenesis of AF is not new, but there is still uncertainty about its relation to the clinical presentation and course of the disease.

Logically this raises the question whether the inflammatory response precedes the manifestation of the rhythm disorder or is a consequence of it. The design of the study does not allow for a definitive answer. The specific nature of the results, however, namely the significantly increased plasma concentrations of hs-CRP and SAA and the lack of change in the levels of fibrinogen, is an evidence that the changes in the concentrations of the APPs have occurred very recently in time. This in turn is a major prerequisite to assume that they are directly related to the manifestation of the rhythm disorder and are not an accidental laboratory result.

The studies carried out so far on the acute-phase response in patients with PAF present conflicting results. Most often they examined the plasma levels of hs-CRP and SAA. Some authors found significant increases in these parameters in the manifestation of PAF, as well as in unsuccessful cardioversion [19-22]. Based on these results, they conclude that inflammation plays an important role in the manifestation and retention of PAF [23]. Chung et al even suggested that CRP levels may be determined by the “burden” of AF [24, 25]. Other authors, however, did not detect changes in the APPs [26-29]. These discrepant results give us a reason to conclude that the active inflammatory response found in some studies is not a consequence of the rhythm disorder itself, but is associated with the demographic and clinical characteristics of patients. Therefore, a great number of diseases, associated with increased inflammatory activity, were exclusion criteria for our study (see exclusion criteria). Moreover, the control group was formed as identical to the patient group in a number of factors known to affect the concentrations of APPs, namely, sex, age (in decades), BMI, harmful habits, comorbidities and their treatment in order to eliminate their impact on the APPs values.

The results obtained 24 h after the farmacoversion are of particular interest. The trends observed during the clinical manifestation of the disease persist after the restoration of sinus rhythm. The values of hs-CRP and SAA remained elevated (P < 0.05) (Figs. 1, 2), while the levels of fibrinogen have again no statistically significant difference compared to the controls (P > 0.05) (Fig. 3). The results are extremely important because they show that even in sinus rhythm there is an activated response to APPs. However, the lack of change in the values of fibrinogen is a prerequisite to assume that the manifestation of PAF is associated with a low-grade recent-onset inflammatory process. The twice measured elevated plasma concentrations of hs-CRP and SAA undoubtedly prove that inflammation is not just a momentary phenomenon in the clinical course of the rhythm disorder. Its effect on the body, and in particular on the electrophysiological and structural remodeling of the atria, continues even after the regularization of rhythm. The exact mechanisms through which inflammation is involved in the development of AF are not yet established. It is known, however, that CRP has the ability to bind to phosphatidylcholine on the membrane of the myocardial cells. This leads to a disorder in the exchange of sodium and calcium ions in sarcolemma vesicles, electrical remodeling of the atria and subsequently to AF development [30]. Naturally, this raises the issue of quantitative relationship between the duration of episodes of increased inflammatory activity and the probability of recurrence of the rhythm disorder.

Twenty-eight days after the sinus rhythm restoration, the values of all three indicators measured in patients did not differ statistically from controls (P > 0.05) (Figs. 1-3). Restoration of sinus rhythm is associated with reduced inflammatory activity and the process develops slowly over time.

It is appropriate to note that in the studies conducted so far, the APPs were mainly measured once. Thus, their major weakness is the inability to follow the inflammatory process over time. In this context, the threefold examination of hs-CRP, SAA and fibrinogen allows us for the first time to follow the dynamics in the inflammatory status of patients with PAF and seek connection with the manifestation of arrhythmia. Sata et al presented a similar study design, according to which, however, the inflammatory indicators were tested only until the 14th day after the restoration of sinus rhythm [31]. The short observation period does not allow the authors to identify dynamic changes. The elevated levels of hs-CRP persisted until the end of the study, which in turn raises the question whether the inflammatory activity is associated with the manifestation of rhythm disorder or is an accompanying finding.

**Figure 5:**
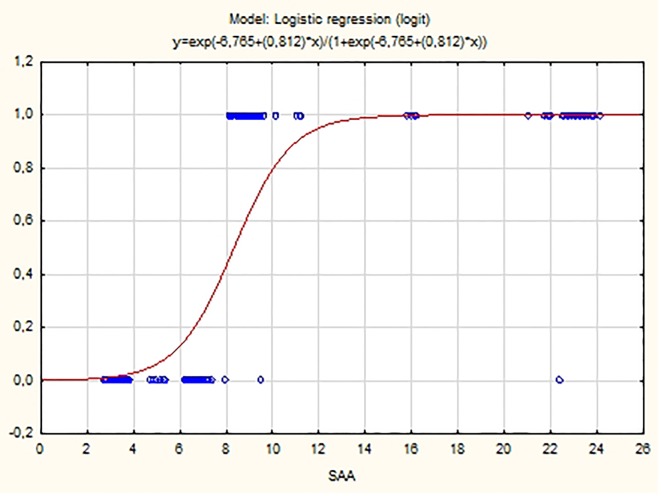
Probability distribution of SAA estimated using logistic model.

Several studies found that the values of hs-CRP and SAA could be predictive of the clinical manifestation of PAF. Dernellis et al assessed the CRP levels in patients with recent-onset AF (< 24 h) and control subjects matched by age, sex and risk factors. They found out that the protein level is a potent determinant of successful cardioversion [19]. In a study by Cheng et al, hs-CRP and SAA levels also correlate with the clinical manifestation of the disease [21]. Our results confirmed the predictive value of these indicators. The logistic regression analysis found that the values of the APPs hs-CRP and SAA are predictive for the clinical manifestation of PAF (P = 0.01 and P < 0.001, respectively) (Figs. 4, 5). The elevated concentrations of the tested indicators are associated with an increased probability of manifestation of the rhythm disorder. These results could have significant clinical applications. They give grounds to use the levels of hs-CRP and SAA in clinical practice as a part of a comprehensive assessment of the need for anti-recurrence treatment.

### Conclusion

In conclusion, we could say that the clinical manifestation of PAF is associated with an inflammatory response characterized by specific dynamics in the plasma concentrations of the APPs hs-CRr and SAA. Their elevated values measured still in the early hours of the rhythm disorder (around the eighth hour). They persist after the sinus rhythm restoration and subside slowly over time. The characteristic features of these changes suggest that the response of the APPs is closely related to the initiating mechanisms of PAF.
